# Understanding the implications of the EU-LULUCF regulation for the wood supply from EU forests to the EU

**DOI:** 10.1186/s13021-018-0107-3

**Published:** 2018-10-16

**Authors:** Gert-Jan Nabuurs, Eric J. M. M. Arets, Mart-Jan Schelhaas

**Affiliations:** 1 Wageningen Environmental Research, Wageningen University and Research, Wageningen, The Netherlands; 2 Chairgroup Forest Ecology and Forest Management, Wageningen University and Research, Wageningen, The Netherlands

**Keywords:** Carbon sink, Sequestration, European forests, EFISCEN, Forest management, Wood mobilisation

## Abstract

**Background:**

In June 2018, the European Parliament and Council of the European Union adopted a legislative regulation for incorporating greenhouse gas emissions and removals from Land Use, Land Use Change and Forestry (EU-LULUCF) under its 2030 Climate and Energy Framework. The LULUCF regulation aim to incentivise EU Member States to decrease greenhouse gas emissions and increase removals in the LULUCF sector. The regulation, however, does not set a target for increasing the LULUCF carbon sink, but rather includes a ‘no net debit’ target for LULUCF (Forests and Agricultural soils). For Managed Forest Land (MFL) an accounting framework with capped credits for additional mitigation against a set forest reference level (FRL) was agreed for 2021–2030. The FRL gives the projected future carbon sink in the two compliance periods 2021–2025 and 2026–2030 under “continuation of forest management practices as they were in the reference period 2000–2009”. This FRL was disputed by some Member States as it was perceived to put a limit on their future wood harvesting from MFL. Here we simulated with the EFISCEN European forest model the “continuation of forest management practices” and determined the corresponding wood harvest for 26 EU countries under progressing age classes.

**Results:**

The simulations showed that under “continuation of forest management practices” the harvest (wood removals) in the 26 EU countries as a whole can increase from 420 million m^3^/year in 2000–2009 to 560 million m^3^/year in 2050 due to progressing age classes. This implies there is a possibility to increase absolute wood harvests without creating debits compared to the forest reference level. However, the manner in which ‘continuation of forest management’ developed with a progressing age class development over time, meant that in some countries the future harvesting exceeded 90% of the increment. Since this generally is considered to be unsustainable we additionally set a harvesting cut-off as max 90% of increment to be harvested for each individual country as a possible interpretation of sustainability criteria that are included in the regulation. Using this additional limit the projected harvest will only increase to 493 million m^3^/year.

**Conclusions:**

The worry from Member States (MS) that the FRL will prevent any additional harvesting seems unwarranted. Due to differences between Member States concerning the state of their forest resources, the FRL as a baseline for harvesting works out very differently for the different Member States. The FRL may have other unforeseen consequences which we discuss. Under all scenarios the living forest biomass sink shows a decline. This can be counteracted through incentivising measures under Climate Smart Forestry.

## Background

It is not disputed that global forests play a large role in regulating the Earth’s climate [1, 2]. However, how to account for this role within global legal agreements distinguishing the additional role that humans can achieve, has appeared a daunting task. Already in the negotiations leading up to the Kyoto Protocol in 1997, concerns about the consequences of incorporating the total existing forest sink in the climate targets had the policy outcome of imposing significant limits on accounting the role of forests in climate change mitigation efforts [3]. The concerns were that when an existing sink would simply be included in the accounted efforts, the measures to limit the use of fossil fuels would be delayed and thus the root cause of climate change not be tackled. Moreover, including the existing forest sink, which may even be increasing due to tree age related developments, does not reflect real efforts for increasing carbon removals. Finally, also the inherently large uncertainty associated with the land use, land-use change and forestry (LULUCF) activities contributed to the felt need for limiting the contribution of forest management to the accounted achievement of reduction targets.

The accounting of mitigation achievements under the Kyoto Protocol includes two commitment periods in which the accounting of Forest Management has evolved from voluntary accounting with a capped amount under the first commitment period^1^ to compulsory accounting against a forest management reference level (FMRL) in the second commitment period (CP2) which ends by the end of 2020 (see [4] for a detailed assessment). To prevent large amounts of credits to become available from forest management, also in the CP2 potential credits from forest management are capped at a maximum amount (3.5% of a party’s total net base year emissions, where base year in most cases refers to 1990).

Under the Paris Agreement countries pledge ambitious climate mitigation targets in their Nationally Determined Contributions (NDC). In these NDCs still high expectations for forest derived mitigation emerge; forests are assumed to provide up to a quarter of planned emission reductions by 2030 [5], in rapid decarbonisation scenarios [6], and in estimates of land-based mitigation potential [7]. Globally, most of the cost-effective mitigation potential is expected from avoided deforestation in the tropics. However, the management of temperate and boreal forests provides many options of effective mitigation as well e.g. [8] including using wood-based products and bio-energy.

EU forests have contributed to climate mitigation already for decades because they have been accumulating more timber volume (growing stock) than was harvested [9]. For the period 2000–2016, they acted as an average net sink of ≈ 430 Mt CO_2_/year, equivalent to about 9% of total EU GHG emissions over the same period [10]. Most of this sink (≈ 380 Mt CO_2_/year) occurs in the “Forest Land remaining Forest Land” category (which is the same as the Managed Forest Land under the new accounting regulation), with the remainder in the “land converted to forest” (including afforestation or reforestation) category. Since forests are getting older in most EU countries, and because older forests grow more slowly, the extent to which this sink may be sustained in the near future is uncertain [9].

Compared to the strictly defined accounting and reporting rules under the Kyoto Protocol, the Paris Agreement leaves a larger degree of freedom for the parties in developing their accounting systems. However, given the earlier voiced concerns over incorporating an existing sink in the reduction targets, the UN laid out principles over when countries “account” for the impact of mitigation actions towards their NDCs (including the forest sector), i.e. they “shall promote environmental integrity, transparency, accuracy, completeness, comparability and consistency, and ensure the avoidance of double counting” (Art 4.13 of the Paris Agreement).

In response to this, in 2016 the European Commission presented a proposal for a regulation on accounting the LULUCF sector within the EU’s 2030 Climate and Energy Framework. The Climate and Energy Framework aims to achieve by 2030 a total emission reduction of 40% relative to 1990 for all sectors together [11]. The inclusion of LULUCF in the 2030 Climate and Energy Framework aims to incentivize EU Member States to decrease greenhouse gas emissions and increase removals in the LULUCF sector. The Regulation, however, does not set a target for increasing the LULUCF carbon sink, but rather includes a ‘no debit’ target for LULUCF (forests and agricultural soils) that should ensure that within the LULUCF sector accounted emissions from land use are entirely compensated by an equivalent removal of CO_2_ from the atmosphere. If this “no-debit” rule is not met within LULUCF in a country, than emissions from LULUCF will need to be compensated by extra emission reduction in other GHG sectors.

After a legislative process that included negotiations among Member States for the European Council’s position on the regulation, similar discussions within the EU parliament and finally negotiations among Commission, Council and Parliament, Regulation 2018/841 was published in June 2018 [12]—referred to here as LULUCF regulation.

An important element in the accounting rules in the LULUCF regulation is that, similar to accounting of forest management under CP2 of the Kyoto Protocol, the mitigation achievements of Managed Forest Land are determined against a forest reference level (FRL), however now against a more strictly described FRL, trying to avoid the large variation in the manner in which MS set their reference level under the Kyoto Protocol. Again this should remove increased carbon removals (accounted as credits) due to age developments of trees and forest that can be expected without any additional efforts from the accounting, making the accounting more similar with other sectors (see [13] for detailed reasoning and examples). An important difference with the accounting against the FMRL in the Kyoto Protocol’s CP2 is that in the projections of future forest developments and harvesting under the FMRL current and foreseen policy developments were included, while in the projections of the FRL the future impact of existing and future policies (like expected increasing demand for wood to meet bio-energy needs) is not taken into consideration. If such additional wood harvests from planned policies or expected demand would already be included in the projections of the FRL, the projected (CO_2_) removals under this FRL would decrease. If then these additional wood harvests are realised during the compliance period, the associated reduction in CO_2_ removals is already discounted by the lower FRL and consequently will thus not be accounted in the LULUCF sector [13], where they normally are accounted. This, because the emissions from biomass burning for energy purposes are not accounted in the energy (ETS) sector [13] provides further detailed reasoning why excluding existing and foreseen policies in the FRL projections is important for the credibility of the FRL approach.

Setting such a forest reference level, however, may mean that any desired harvest increase for a bio-economy may be limited in the future if countries take carbon debits serious. This is because in the short term increased harvesting of trees will reduce the CO_2_ removal capacity of the existing forests, even though a (reduced) sink still exists. Depending on its eventual use, part of the additional harvest will result in increased carbon storage in the harvested wood products pool, which needs to be included in the FRL emissions and removals. Increasing the share of wood in products with a long life span compared to the reference period then will cancel out some of the losses occurred in Managed Forest Land.

In the negotiations between Member States and with the Commission the rules for setting this FRL and possible compensation of future debits has been a major obstacle, also because calculations of different options for a reference level were not available. Particularly interpreting the consequences of projecting management practice from the reference period has resulted in confusion and misunderstanding. While by some this was translated as keeping levels of wood harvesting from the reference period constant [14], this is not what the regulation asks for [15]. Instead, the wood harvests considered in the projections for the reference level depend on the more autonomous development of biomass (or growing stocks) as a result of age dependent growth. If areas and growing stocks of available biomass are projected to increase during the compliance period of 2021–2030, so will the actual wood harvest potentials as projected under the FRL. Real observed carbon stock changes in managed forests in the periods 2021–2025 and 2026–2030 will then be compared against this FRL. Any increases in carbon removals from Managed Forests that are greater than the FRL will be counted as credits, while decreases in carbon removals from Managed Forests will result in debits.

### Aim

Here we simulated with the EFISCEN European forest model the “continuation of forest management practices” and determined the corresponding wood harvest for 26 EU countries under progressing age classes. The aim of this study was to assess the likely consequences of the LULUCF regulation on the volumes of wood coming available to the EU from EU forests by projecting future forest characteristics and developments under continuation of forest management practice from the reference period and calculating the corresponding developments in harvesting levels from Managed Forest Land for EU Member States. Managed Forest land is Forest land that has been Forest land for at least 20 years (see the EU LULUCF regulation and definitions therein). Applying the continuation of sustainable forest management practice from the reference period in a consistent manner for all EU countries (except Malta, Cyprus), we aimed to assess the limits which may arise in future roundwood harvesting levels assuming countries will wish to avoid debits.

## Methods

We applied the European Forest Scenario Model (EFISCEN), a forest resource model to calculate three scenarios of interpretations of the LULUCF regulation text). The European Forest Information SCENario Model (EFISCEN) is a large-scale forest model that projects forest resource development on regional to European scale (see efiscen.efi.int and [16–20]). It uses national forest inventory data as a main source of input to describe the current structure and composition of European forest resources. EFISCEN is a matrix model, where the state of the forest is represented in matrices as an area distribution over age and volume classes. Aging is simulated as the movement of area to higher age classes, while growth is simulated as the movement of area to higher volume classes. Thinning is simulated as movement of area to a lower volume class, while the difference in volume is assumed to be the volume that has been removed by the thinning. Final felling is simulated by moving the area back to the first volume and age class of the matrix from where it can start growing again. The volume originally present at this area is the volume removed during final felling.

Harvest regimes are specified at two levels in the model. First, a basic management regime per forest type and country defines the age range during which thinnings can take place and a minimum age for final fellings. These regimes can be regarded as constraints on the total harvest level. Multiplication of the area available for thinnings and final fellings with the corresponding wood harvest gives the amount of wood that is theoretically available for harvesting. In the second step, the actual demand for wood is specified for thinnings and for final felling separately at the national level. The model calculates which share of the available potential needs to be harvested to satisfy the demand and implements this calculated intensity in the simulation.

EFISCEN is a rather versatile European forest resource model providing detailed insights down to NUTS2 level and up to European scale. It has been applied in studies concerning impacts of management changes [16], or to include impacts of climate change and its resulting carbon balance [21, 22]. Later on also for upscaling effects of natural disturbances and impacts of adaptive management [18] or for wood availability and trade-offs with biodiversity [19]. The model’s latest version is documented in [20].

### Three harvesting scenarios

In Scenario 1 we interpreted the LULUCF text in a manner as was discussed extensively amongst Member States, namely that the LULUCF regulation would limit the harvesting at constant absolute amount of wood over time. In this scenario the harvest level per time step is derived from the actual wood production as extracted from the FAOSTAT database where we applied the average harvest as observed in the period 2000–2009. We regard this as a baseline scenario to derive a forest ecosystem carbon sink development over time. This is called ‘*constant absolute amount of harvest’.*

**Fig. 1:**
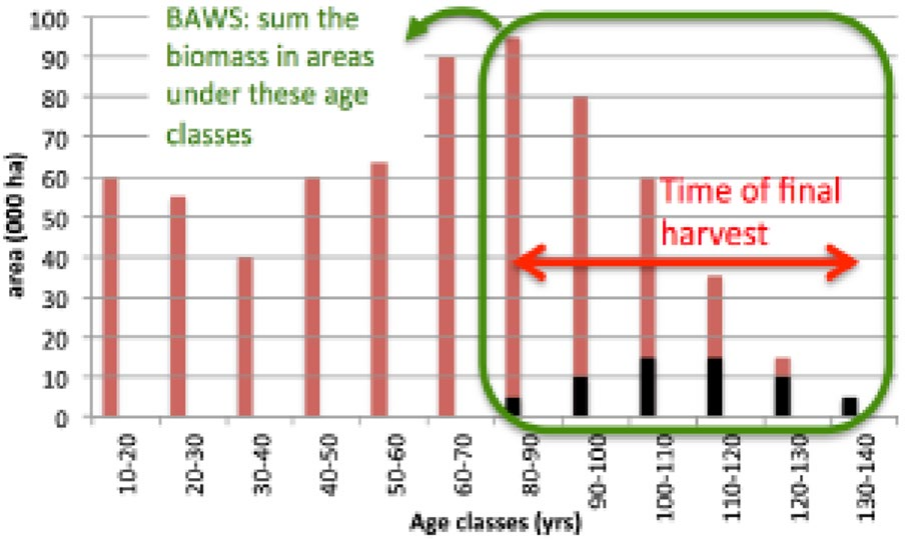
Hypothetical forest age class distribution of a country. The green encircled part are those age classes where under the base period harvesting took place. The biomass associated to these is called ‘BAWS’, biomass available for wood supply. The black part of the bars are those areas that have actually been harvested between 2000 and 2009. It is these black fractions out of the red bars, that together form the management fraction. This percentage is then used in the projection under ‘constant management intensity’ [23]

In Scenario 2, we calculated the harvest fraction in the first time step (corresponding to the base period 2000–2009) and applied this throughout the rest of the simulation as an interpretation of Article 8.5 of the Regulation “The forest reference level shall be based on the continuation of sustainable forest management practice, as documented in the period from 2000 to 2009 with regard to dynamic age-related forest characteristics in national forests, using the best available data”. In the simulation the amount of wood harvested over time is thus the result of a fixed continuation of management and changes in the state of the forest over time (Fig. 1). We regard this approach as the most in line with the LULUCF regulation [23, 24]. This is called *‘constant intensity’.*

In Scenario 3, the amount of wood harvested from scenario 2 is applied as demand, but when running scenario 2 it sometimes lead to a national harvest rate of more than 100% of the increment (due to fast ageing of the forest). However Annex IV (of 2018/841) states that criteria for determining reference levels are: ‘..consistent with the objective of contributing to the conservation of biodiversity..’. Furthermore the forestry accounting plan shall contain ‘..documentary information on sustainable forest management practices and intensity and adopted national policies’. We have interpreted these as that a harvesting level of more than 100% of the increment would not be acceptable. In forestry practice a felling level of a maximum of 90% of the increment is a rather widely accepted and pragmatic sustainability principle. This we have included in the simulations here. This is called ‘*constant intensity plus cut off at sustainable level’.*

The initialisation data are the same as used in the EFSOS II study [25]. Simulations and harvest regimes (roundwood removals overbark) are based on the EFSOS II baseline scenario with some updates from the Volante project [26] yielding a total net annual increment of 789 million m^3^/year in 2015 (see Appendix).

## Results

**Fig. 2:**
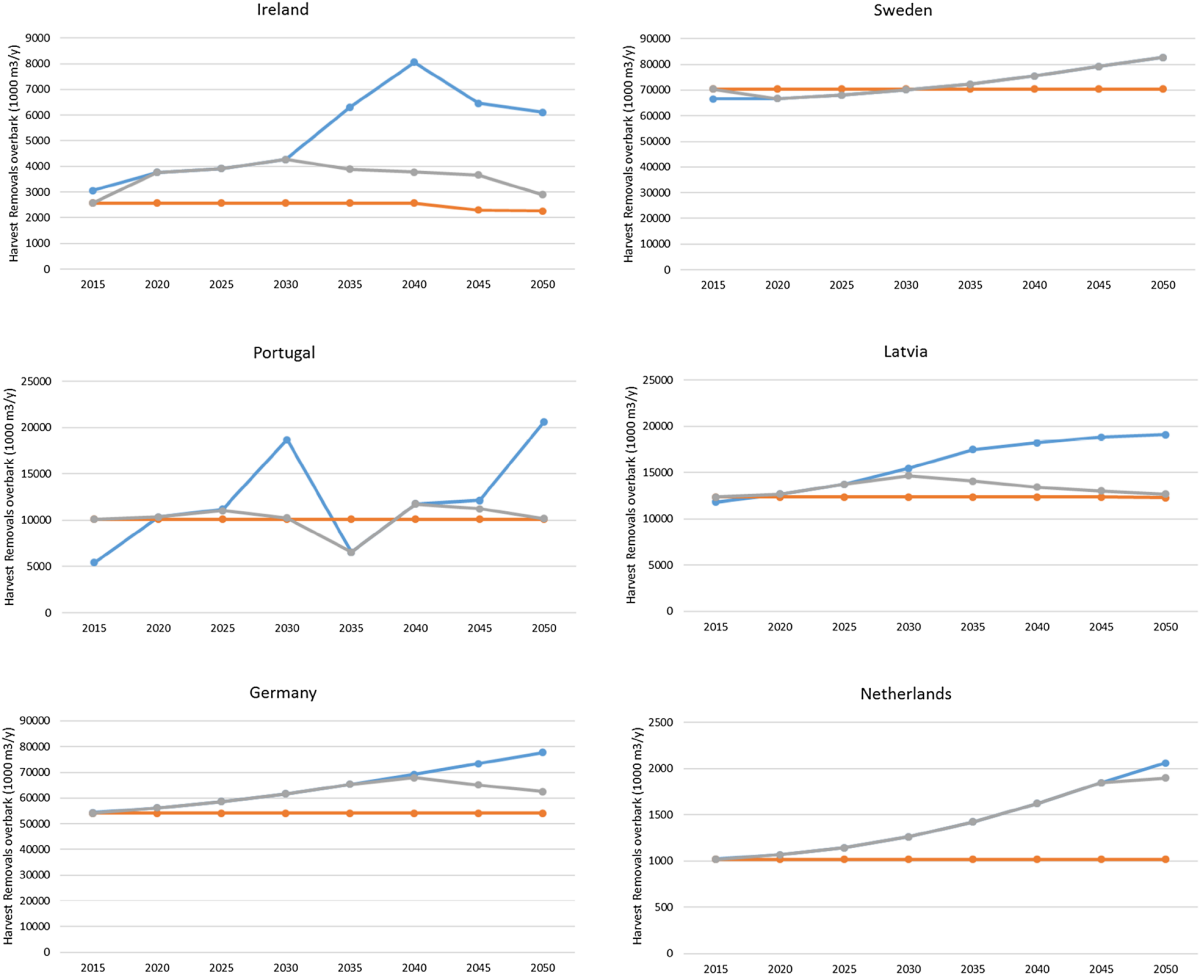
Development of potential annual harvest (removals overbark) until 2050 under the forest reference level without creating debits for selected EU countries under the three scenarios of the LULUCF regulation text. Orange line: constant absolute amount of harvest, blue line: constant intensity, grey line: constant intensity plus cut off at sustainable level

**Fig. 3:**
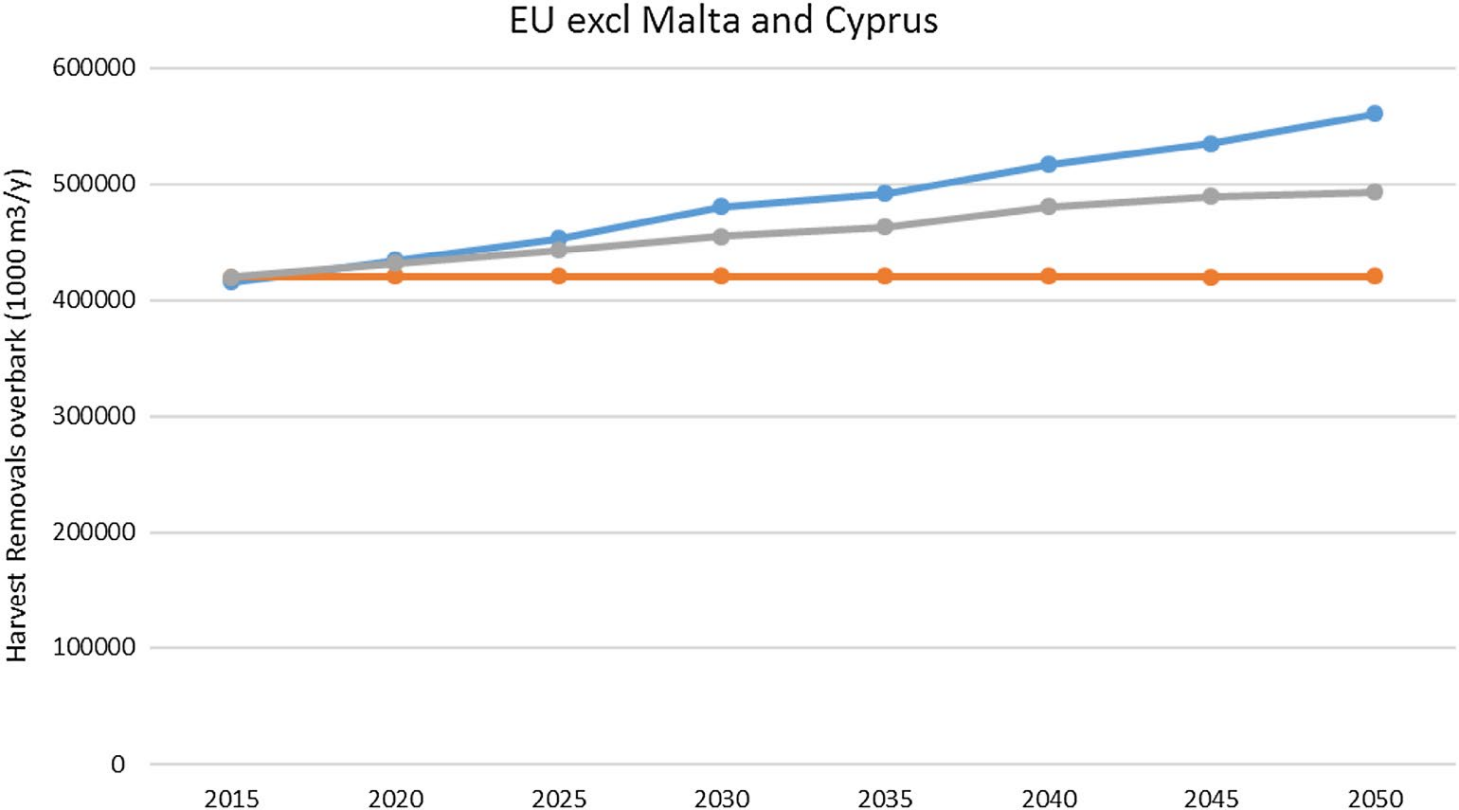
Development of potential annual harvest (removals overbark) until 2050 under the forest reference level without creating debits for all EU countries (excluding Malta and Cyprus) under the three scen of interpretations of the LULUCF regulation text

Here we present results for six exemplary countries and the EU as a whole (excluding Malta and Cyprus), not assuming any future growth changes due to e.g. climate change or improved forest management. Figure 2 shows the reference level harvest under three scenarios of alternative interpretations of the Regulation text through which a reference level sink shall be determined for selected countries. In total 26 countries were run, but here only six are displayed that represent the diversity of outcomes. Higher harvest than this would result in debits, and depending on credits and debits in other activities would set the compensation mechanism of the LULUCF regulation into action. Figure 3 then gives the total EU26 harvest development.

Depending on age class structure and historic (2000–2009) management practices, most countries and the EU as a whole show an increase in absolute harvest under continuation of management of scenario 2, following gradual aging of the forest resource over time. Thus the MS’ worry that the EU regulation would set an absolute maximum limit on harvest at the level where it is today, is not supported by these runs. The EU 26 as a whole shows a harvest removals increase from 420 million m^3^ in 2000–2009 to 560 million m^3^ in 2050, complying to management practices criteria. If we however also set the cut-off to comply to a pragmatically chosen sustainability criteria at a maximum of 90% of increment to be harvested for each individual country (scenario 3), then the harvest can only increase to 493 million m^3^/year in 2050. The felling/increment ratio then becomes 80% for the EU26 as a whole, with values for individual countries ranging between 29 and 90% from now to 2050.

However, single countries show very different patterns over time. Ireland for example has planted a lot of forest over the last decades and shows almost a doubling of its absolute harvest volume under a continuation of management practices until 2035. Whereas Sweden shows only an 8% increase. The other countries in the examples show between 20 and 47% increase in harvest.

However, under this constant intensity, harvest levels will in some cases be temporarily much higher than the increment, so the sustainable scenario yields a much lower sustainable harvest level. The Irish, Latvian and to lesser extent German case would be limited by this sustainability limit. Sweden is the only example where the 90% sustainability limit does not affect the simulated potential harvest level.

**Fig. 4:**
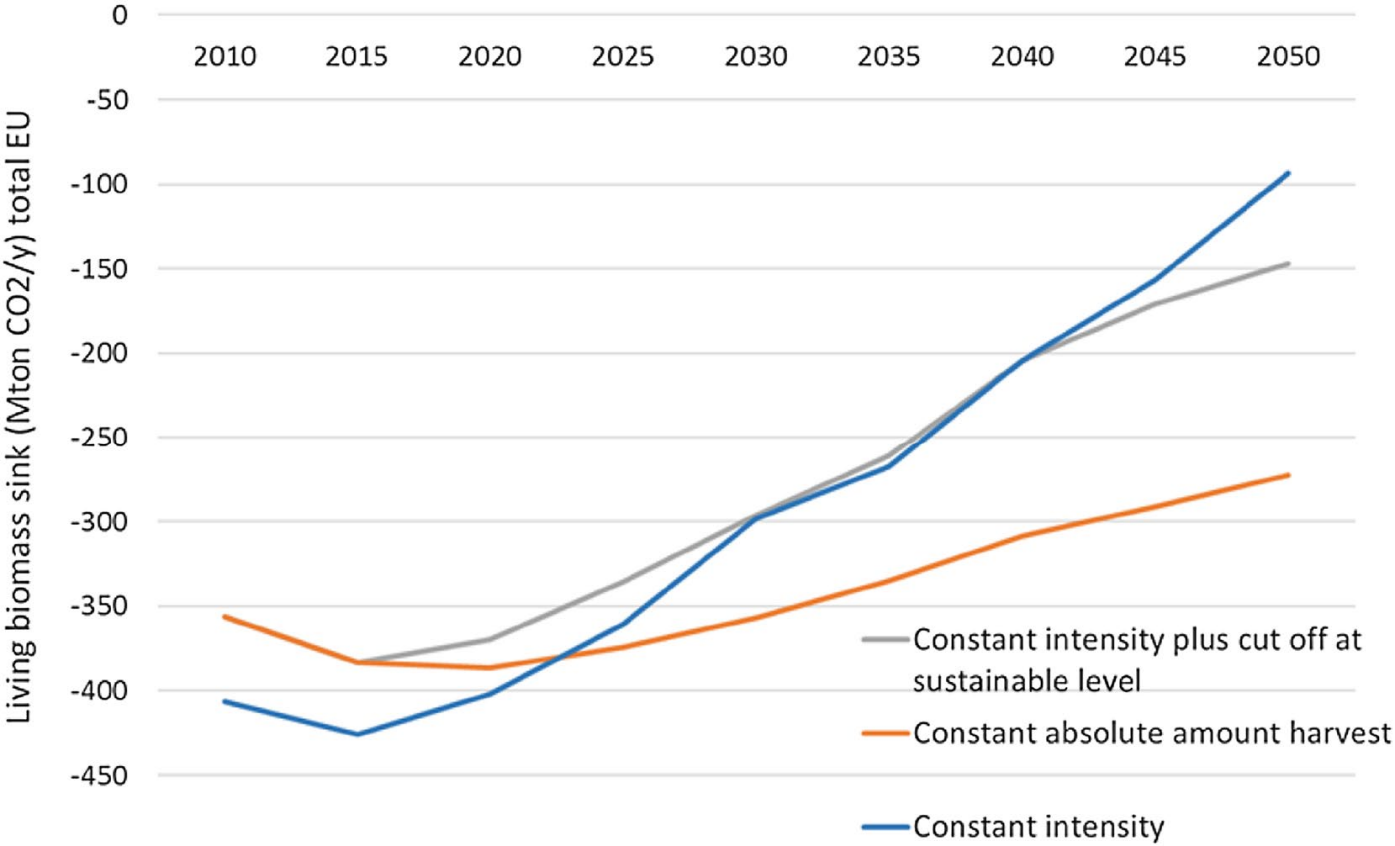
Living biomass sink development for total EU26 forests under the three scenarios. The initial sink in 2010 is larger (more negative) under ‘constant intensity’ because the runs start in 2005 and harvesting levels vary for 2010

In Fig. 4 the sink development is given for the three scenarios. Under all scenarios the sink declines. The ‘constant intensity’, with the highest harvest, shows the most decline. It declines from current − 430 million tonnes CO_2_/year to − 298 million tonnes CO_2_/year in 2030, not assuming any impacts of climate change or other management changes.

## Discussion

The current study findings are important in the sense that one consistent modelling approach with three scenarios of interpretation of the text of the LULUCF regulation was used. This provides insights in how the regulation may work out in terms of harvesting if countries don’t want debits. In [13] such results based on runs with the CBM model (which is partly parameterised with EFISCEN data) were also presented, but they mostly presented the reasoning behind the LULUCF regulation, and its technical and scientific approach for credible accounting. They show only EU level results which under a no-debit assumption provides a harvest removals increase from 500 million m^3^/year in the 2010s to 550 million m^3^/year in 2030; the trend being much in line with our scenario 2 results.

Another study regarding effects of reference levels on the European forest sector was presented by [14]. They used a different approach with the market model EFI-GTM. They conclude that harvests will be reduced with 119 million m^3^/year by 2030 due to the Regulation compared to a “baseline without the Regulation” assuming a steady increase in EU wood demand over time. In their study export/import is directly considered. A harvest constraint (as assumed by them) and imposed on the EU due to the Regulation would lead to higher roundwood prices in the model, and this results in the trade model in changed trade. In EFI-GTM demand then shifts to other regions through trade. The simulated changes in roundwood prices then negatively influence the EU forest industry production, further reducing demand for raw material and thus the harvest. This study was later commented on by [15] stating that the upfront reduction of harvesting as assumed by [14] was a wrong assumption.

Some reservations to the current study are also warranted. Even though EFISCEN is a well-established versatile forest and carbon tool designed for European forests, National Forest Inventory input data did not always represent the situation of 2000 (Appendix). The management intensity was thus not always calculated exactly for the period 2000–2009. However, age class distributions do not change very quickly [18] and the deviations are expected to be only minor. Furthermore, increments are not always up to date; the same is valid for the area of forest available for wood supply (Appendix).

Another uncertainty affecting all studies is that EU harvesting levels are rather uncertain. We can state that most European States have a solid forest inventory [27], representing the state of the forest resource very well, there is still large uncertainty over harvesting levels. For many countries the statistics from [27] or FAOSTAT have their shortcomings. For some countries there are very large differences between the reported periods, and sometimes data are corrected in later versions. E.g. [28] (through a wood resource balance) report that for the EU as a whole there are some 98 million m^3^ of “missing” sources, and to a considerably extent, they are the result of unreported (harvest) removals. The reason why [13] has a higher harvesting level is that they have corrected for these underestimated harvests. These same data problems valid for our study, however will also apply to [13].

Even though we found in the present study that countries are likely permitted additional harvesting in their FRL, the placement of some sort of quota system on harvesting may have unintended consequences. E.g. larger additional forest resource use as projected under the bio-economy case of Finland and Sweden may in the short-term lead to a strongly reduced sink even though they will continue to have a net sink. Thus they may be debited for a transition towards a more sustainable (free of fossil fuels) future. This worry about a future debit, may hamper this transition.

Furthermore, if this Regulation is perceived as a quota system on harvesting, it may dis-incentivise forest owners to invest in their forests. On the other hand: how much influence will one Regulation really have? Management actions on European forests are carried out by more than 16 million private owners and thousands of public owners [27]. It is also clear that despite enormous changes in society over the last six decades, harvest levels at EU level have remained relatively stable. Thus, this large resource acts as a body with a very large inertia, apparently rather insensitive to incentives from outside. Furthermore the Regulation certainly provides the possibility to stimulate and invest in forest resources and forest expansion, leading to higher increment and finally harvesting as well, following Climate Smart Forestry [8]. The big question is if countries will take harvesting under the forest reference level as some sort of quota system and how serious they will take debits. If they do perceive a supply limit, it will drive up raw material prices even if there is not a real shortage to meet demand. If the Regulation is perceived as a ceiling to supply, then it is quite well possible that the future bio-economy industry will look at other continents, leading to less investments in EU forests.

## Conclusions

We quantified the harvesting possibilities under the LULUCF regulation, provided a country does not want to generate debits. The simulations showed that the EU 26 as a whole may have a harvest (wood removals) increase from 420 million m^3^ in 2000–2009 to 560 million m^3^ in 2050, complying to ‘continued management practices’ criteria, without creating debits. However, another unexpected finding came out of this study as well. The manner in which ‘continued management practices’ works out with a progressing age class development over time, means that in some countries the harvesting exceeds 90% of the increment. When we set a cut-off to comply to a practical sustainability criteria of 90% of the increment to be harvested, then the harvest can only increase to 493 million m^3^/year in 2050. The removal/increment ratio then becomes 80% for the EU26 as a whole, with values for individual countries ranging between 56 and 90%.

Under all scenarios the living biomass sink shows a decline. It declines from current − 430 million tonnes CO_2_/year to − 298 million tonnes CO_2_/year in 2030 under the ‘constant intensity’ scenario, not assuming any impacts of climate change. If Member States want to avoid this saturation they would have to implement additional measures that are certainly allowed under the Regulation (next to higher harvest) in line with Climate Smart Forestry.

## Appendix

See Table 1.

**Table 1:** Metadata of EFISCEN runs

	Base year of forest inventory	Area included in EFISCEN’s FAWS 2010 (kha)	SOEF 2015 area FAWS for 2010	Net annual increment simulated by EFISCEN (1000 m^3^/year)	Net annual increment as given by SOEF 2015 (1000 m^3^/year)
aut	2001–2002	3349	3341	27,763	25,136
bel	1997–1999	587	668	4896	4609
bul	2000	3646	2387	16,648	14,361
cro	1995	1443	1740	5246	8144
cze	2005 (2015)	2712	2310	27,343	20,463
den	2000	473	552	5035	6263
est	1999–2001	2048	2008	11,047	11,514
fin	2004–2008	18,551	19,465	94,542	93,379
fra	1988–2000	13,873	15,607	102,445	82,871
ger	2001–2002	9781	10,886	104,864	118,589
gre		3595	3595	4673	–
hun	2005	1859	1729	13,308	9774
ire	2004–2005	626	608	6226	6677
ita	2005–2008	8759	7979	33,109	32,543
lat	2004–2008	3141	3149	21,121	19,680
lit	200	1939	1852	10,717	11,030
lux	1989	71	86	741	650
nla	2001–2005	360	299	3008	2738
pol	1993	6309	8128	33,164	62,300
por	1997–1998	2027	2147	14,458	18,870
rom	1980s	5643	5147	48,832	29,260
slo	2000	1159	1139	7790	9165
slr	1994	1909	1779	11,839	13,465
spa	1986–1995	10,473	14,574	27,014	35,479
swe	2004–2008	22,647	20,033	119,377	96,486
uka	1995–2000	2094	3059	16,871	23,113
Total		130,213	135,460	785,524	765,560

*SOEF* State of Europe’s Forests [27], *FAWS* area of forest available for wood supply

## Abbreviations

BAWS: biomass available for wood supply
EFSOS: European Forest Sector Outlook Study
FMRL: forest management reference level (under the Kyoto Protocol)
FRL: forest reference level
LULUCF: land use, land-use change and forestry
MS: Member States
NDC: Nationally Determined Contributions

## Footnotes

1 The accounting rules for Forest Management in CP1 were agreed on in paragraphs 10 and 11 of Article 3.4 in the Annex to UNFCCC Decision 16/CMP.1 (http://unfccc.int/resource/docs/2005/cmp1/eng/08a03.pdf).
